# TransferBAN-Syn: a transfer learning-based algorithm for predicting synergistic drug combinations against echinococcosis

**DOI:** 10.3389/fgene.2024.1465368

**Published:** 2025-01-06

**Authors:** Haitao Li, Yuanyuan Chu, Liyuan Jiang, Lei Li, GuoDong Lv, Yuansheng Liu, Chunhou Zheng, Yansen Su

**Affiliations:** ^1^ Key Laboratory of Intelligent Computing and Signal Processing, School of Artificial Intelligence, Anhui University, Hefei, China; ^2^ Institute of Artificial Intelligence, Hefei Comprehensive National Science Center, Hefei, Anhui, China; ^3^ State Key Laboratory of Pathogenesis, Prevention, and Treatment of Central Asian High Incidence Diseases, Clinical Medical Research Institute, First Affiliated Hospital of Xinjiang Medical University, Urumqi, China; ^4^ College of Computer Science and Electronic Engineering, Hunan University, Changsha, China

**Keywords:** echinococcosis, drug combination, transfer learning, synergistic drug combinations, parasitic diseases

## Abstract

Echinococcosis is a zoonotic parasitic disease caused by the larvae of echinococcus tapeworms infesting the human body. Drug combination therapy is highly valued for the treatment of echinococcosis because of its potential to overcome resistance and enhance the response to existing drugs. Traditional methods of identifying drug combinations via biological experimentation is costly and time-consuming. Besides, the scarcity of existing drug combinations for echinococcosis hinders the development of computational methods. In this study, we propose a transfer learning-based model, namely TransferBAN-Syn, to identify synergistic drug combinations against echinococcosis based on abundant information of drug combinations against parasitic diseases. To the best of our knowledge, this is the first work that leverages transfer learning to improve prediction accuracy with limited drug combination data in echinococcosis treatment. Specifically, TransferBAN-Syn contains a drug interaction feature representation module, a disease feature representation module, and a prediction module, where the bilinear attention network is employed in the drug interaction feature representation module to deeply extract the fusion feature of drug combinations. Besides, we construct a special dataset with multi-source information and drug combinations for parasitic diseases, including 21 parasitic diseases and echinococcosis. TransferBAN-Syn is designed and initially trained on the abundant data from the 21 parasitic diseases, which serves as the source domain. The parameters in the feature representation modules of drug interactions and diseases are preserved from this source domain, and those in the prediction module are then fine-tuned to specifically identify the synergistic drug combinations for echinococcosis in the target domain. Comparison experiments have shown that TransferBAN-Syn not only improves the accuracy of predicting echinococcosis drug combinations but also enhances generalizability. Furthermore, TransferBAN-Syn identifies potential drug combinations that hold promise in the treatment of echinococcosis. TransferBAN-Syn not only offers new synergistic drug combinations for echinococcosis but also provides a novel approach for predicting potential drug pairs for diseases with limited combination data.

## 1 Introduction

Echinococcosis is a zoonotic parasitic disease caused by the larval stages of tapeworms of the genus echinococcus, primarily affecting organs such as the liver and lungs (Alvi et al., 2023; Meng et al., 2023). This disease manifests in two forms: Cystic echinococcosis and Alveolar echinococcosis, both having significant clinical consequences, potentially resulting in high mortality (Autier et al., 2023; Casulli et al., 2023). The disease is predominantly found in the Mediterranean basin, South America, North Africa, Central Asia, and Eastern Europe, with the western pastoral areas of China being high-prevalence zones (Wen et al., 2019). Alveolar echinococcosis, also known as ‘worm cancer’, is especially dangerous, with untreated cases facing a 10-year mortality rate over 90% percent, significantly impacting the economy of agricultural and pastoral areas (Casulli et al., 2023).

The treatment strategies for echinococcosis include surgical and pharmacological interventions. In the situation that patients cannot undergo surgery, pharmacological interventions are more suitable options (Hogea et al., 2024). Currently available anti-echinococcosis drugs are mainly anti-parasitic drugs and cancer-fighting drugs (Wang et al., 2022). In clinical practice, benzimidazole derivatives, such as albendazole and mebendazole, are widely used to treat echinococcosis (Wen et al., 2019). However, clinical studies have shown that long-term administration of albendazole and mebendazole might cause adverse reactions such as the skin and mucous membranes, nervous system, and cardiovascular system (Qing et al., 2023). Therefore, there is an urgent need to explore effective and safe therapeutic strategies against echinococcosis.

The application of drug combinations in treating various complex diseases, such as cancers and hypertension, is becoming increasingly widespread (Parati et al., 2021; Jaaks et al., 2022). Compared with single-drug treatment, combinations can synergize within biological pathways, enhancing efficacy and hastening recovery, while reducing doses of individual drugs, mitigating potential adverse effects and resistance (Csermely et al., 2013). These improvements enhance the quality of life for patients and reduce discomfort during treatment. Furthermore, combinations can lower the risk of disease resistance, highlighting the importance of identifying effective synergistic pairs in treatment strategies.

Recently, the main strategies to develop anti-echinococcosis drug combinations are based on *in vitro* and *in vivo* experiments.

Loos et al. investigate the *in vitro* anti-echinococcal activity of Octreotide combined with Metformin, demonstrating significant reduction in parasite viability through induced autophagy and upregulation of key autophagic genes, proposing a potential new therapeutic approach for treating cystic echinococcosis (Loos et al., 2020). Mohammadi et al. demonstrate that the combined treatment of Allium sativum methanolic extract with a reduced dose of Albendazole enhances anti-hydatidosis efficacy, achieving similar parasitological outcomes as a higher dose of Albendazole alone, but with reduced hepatotoxic effects (Haji Mohammadi et al., 2019). However, this process is time-consuming and costly. Besides, patients may also be subjected to unnecessary treatment risks (Rani et al., 2022). Moreover, it is difficult to explore all the possible drug combinations solely through biological experiments.

The inefficiency and limitations of this widespread trial-and-error method underline the critical need for more effective experimental evaluation techniques.

In the past decade, machine learning-based methods have played a significant role in the field of drug combination prediction, greatly expanding the capability to explore effective drug combinations (Wu et al., 2022; Liu et al., 2023). For instance, Janizek et al. introduced TreeCombo, which is based on the extreme gradient boosting trees (XGBoost) algorithm to predict the synergy scores of drug pairs (Janizek et al., 2018). Although traditional machine learning-based methods have made progress in drug combination prediction, they still have limitations such as the need for complex manual feature engineering and expertise, as well as insufficient computational power to support large-scale rapid predictions. With the rapid development of deep learning technology and the availability of extensive drug combination data, using deep learning for drug combination prediction has become a new trend. For example, the DeepSynergy model predicts the synergy between drugs by combining their chemical properties and gene expression data from cell lines (Preuer et al., 2018). GAECDS integrates graph autoencoders and convolutional neural networks to predict the synergistic effects of drug combinations (Li et al., 2023). These deep learning approaches not only overcome some limitations of traditional machine learning-based methods but also open new possibilities for exploring and predicting effective drug combinations in cancer (Güvenç Paltun et al., 2021). However, current deep learning approaches still have limitations in predicting synergistic drug combinations for echinococcosis. Firstly, deep learning algorithms for drug combination prediction have primarily focused on cancer due to the extensive genomic data (e.g. gene expression data) available from cancer cell lines, effectively capturing cancer features (Sarmah et al., 2023). Nevertheless, data similar to cancer genomics data is not yet available for parasitic diseases like echinococcosis, which limits the development of computational prediction methods. Secondly, the effective training of deep learning models is contingent upon extensive, high-quality datasets, which should include a vast number of evaluations of drug synergistic combinations (O’Neil et al., 2016). Yet, data on effective synergistic combinations for echinococcosis is exceedingly rare, considerably constraining the training and predictive precision of deep learning approaches. Furthermore, current techniques generally fall short in the integration of features. The current approach is usually to simply concatenate the features of individual drugs without fully considering the possible complex interactions between them. This simple method of combining features ignores the comprehensive effects of drug interactions. Therefore, it cannot reveal the potential interactions between different drug properties. Due to the aforementioned limitations, there are currently no effective algorithms for identifying potential echinococcosis drug combinations.

To overcome these limitations and improve the accuracy of identifying potential synergistic drug combinations for echinococcosis, this study has developed a transfer learning-based framework named the TransferBAN-Syn model. The model, integrating drug combination data from other parasitic diseases, effectively predicts treatment combinations for this disease despite limited existing information. This paper makes three significant contributions:

• We propose a transfer learning-based model, TransferBAN-Syn, to identify synergistic drug combinations against echinococcosis using abundant data from other parasitic diseases. Transfer learning captures valuable knowledge from these other diseases to enhance the performance of the target model in predicting drug combinations for echinococcosis. To the best of our knowledge, this is the first work that employs transfer learning to improve prediction accuracy with limited drug combination data for echinococcosis treatment.

• We constructed a special dataset with multi-source information and drug combinations for parasitic diseases, including 21 parasitic diseases and echinococcosis. The multi-source information includes disease pathway data and disease similarity information, effectively capturing comprehensive disease characteristics. Additionally, utilizing the abundant information from other parasitic diseases helps to enhance the accuracy and generalizability of drug combination predictions for echinococcosis.

• Comparative experiments with traditional machine learning methods (such as TreeCombo (Janizek et al., 2018)) and the latest deep learning models (including DeepSynergy (Preuer et al., 2018), TranSynergy (Liu and Xie, 2021), Attsyn (Wang et al., 2023), GAECDS (Li et al., 2023)) confirm the superior performance of TransferBAN-Syn in predicting the synergistic effects of drug combinations against echinococcosis. Besides, TransferBAN-Syn identifies potential drug combinations that hold promise in the treatment of echinococcosis.

## 2 Materials and methods

### 2.1 Dataset description

#### 2.1.1 Comprehensive parasitic disease drug combination dataset

The comprehensive parasitic disease drug combination dataset collects drug and drug combination information for treating echinococcosis and other parasitic diseases. The dataset is sourced from the China National Knowledge Infrastructure (CNKI) and PubMed databases. For the treatment of echinococcosis, the related drugs and drug combinations include 55 single drugs and 50 drug combinations that have been confirmed to have synergistic effects. Considering the limited information on anti-echinococcosis drug combinations, the dataset also includes 21 other parasitic diseases similar to echinococcosis (see Supplementary Table S1), including 263 single drugs and 283 drug combinations. These 21 parasitic diseases are selected based on their significant biological similarities to echinococcosis. Specifically, these diseases share critical descriptors with echinococcosis, which suggests potential genetic or pathway similarities that may influence their response to drug treatments. The selection process utilizes the Malacards database, where a GeneAnalytics tool analyzes gene-sharing characteristics between echinococcosis and other diseases. Parasitic diseases with a similarity score greater than eight are chosen, as they are likely to exhibit similar responses to drug combinations, making them valuable in supporting the prediction of effective drug combinations for echinococcosis. The efficacy of each drug and its combination is supported by literature. Additionally, drug combinations are identified and organized that have been clearly shown to have no synergistic effects and are unsuitable for use together as negative samples, to enhance the accuracy of the research. This dataset provides a comprehensive and detailed data foundation for the synergistic drug combinations for echinococcosis and related parasitic diseases as shown in Table 1, supporting subsequent research on the identification of potential drug combinations.

**TABLE 1:** Information of drug combination for parasitic diseases.

Disease	Drug	Drug combination		
		Synergy	Non-synergy	Total
Echinococcosis	55	50	100	150
Other 21 parasitic diseases	214	283	243	526
Total	**263**	**333**	**343**	**676**

#### 2.1.2 Multi-source information of parasitic disease

Information from the MalaCards database http://www.malacard.org is utilized to effectively characterize echinococcosis and other parasitic diseases (O’Neil et al., 2016). Malacards is a comprehensive database that provides detailed information on various diseases, integrating data on disease characteristics, associated pathways, clinical features, and related medical conditions. It offers a valuable resource for understanding disease phenotypes and their underlying genetic and clinical aspects. The MalaCards composite relevance score served as the basis for similarity scores between parasitic diseases, uncovering further relevant connections by identifying significant gene overlaps between two diseases, leading to the generation of a composite relevance score (Rappaport et al., 2013). For details on the specific calculation of the MalaCards composite relevance score, see “MalaCards - The Human Disease Database” (https://www.malacards.org/pages/info#disorders).

### 2.2 TransferBAN-Syn model

#### 2.2.1 Overview of transfer learning strategy and TransferBAN-Syn model

The aim of transfer learning is to leverage the source domain and source task to learn the target domain and improve the performance of the target task. The training phase of the transfer learning-based framework usually includes two stages. Initially, a source model is obtained by training the network using a sufficient amount of source training data. This is also known as the pre-trained source model. Then, the pre-trained source model is used as the initial weights and retrained with a small amount of target training data to obtain the target model. The most common transfer learning technique is fine-tuning, which is essentially parameter-based transfer learning. Based on the assumption that the learned parameter values (i.e., weights) from the source domain contain useful knowledge, better performance is achieved by transferring these parameter values to the target model. The parameter values obtained from the source model become the initial values for the target model’s parameters. Thus, the weights of the target model start from the converged values of the pre-trained source model rather than random values. The target model is also retrained with a small amount of target training data and converges faster with fewer training epochs.

**FIGURE 1:**
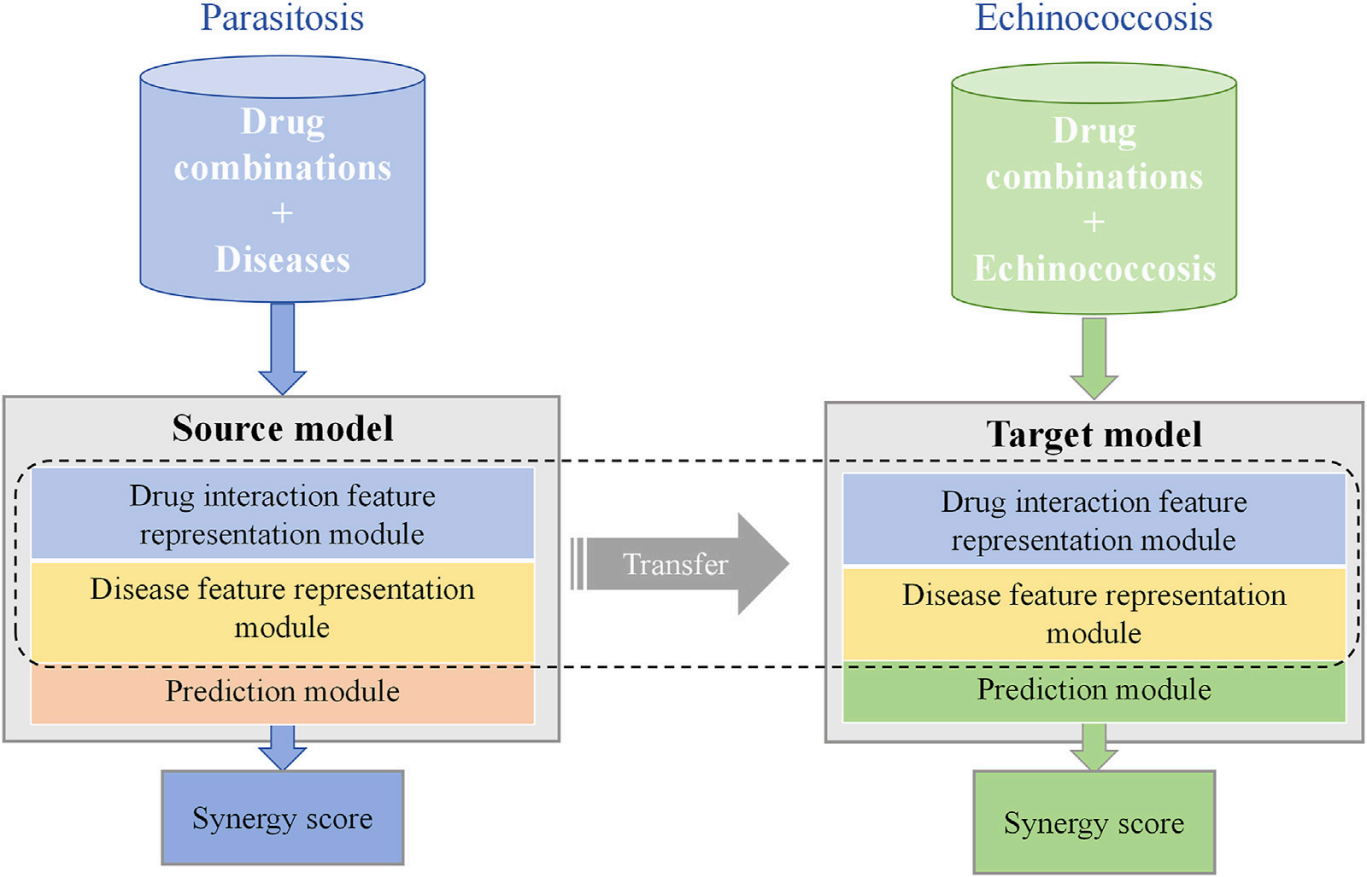
TransferBAN-Syn Transfer Learning strategy. TransferBAN-Syn consists of source domain and target domain models. The source domain model is pre-trained with data-rich parasitic diseases to comprehend the underlying mechanisms between drug combinations and diseases. The target domain model for echinococcosis shares parameters with the source domain model and fine-tunes the prediction module parameters to achieve optimal predictive performance.

The transfer learning-based model, TransferBAN-Syn, employs the predictive knowledge of drug synergistic effects from 21 parasitic diseases to enhance the efficiency and accuracy of predicting drug combinations for echinococcosis (as shown in Figure 1). First, the drug combination prediction model for 21 parasitic diseases is trained, and its parameters are preserved. Then, the target model is trained by preserving the feature extraction parameter and fine-tuning the prediction part, to predict drug combinations for echinococcosis.

**FIGURE 2:**
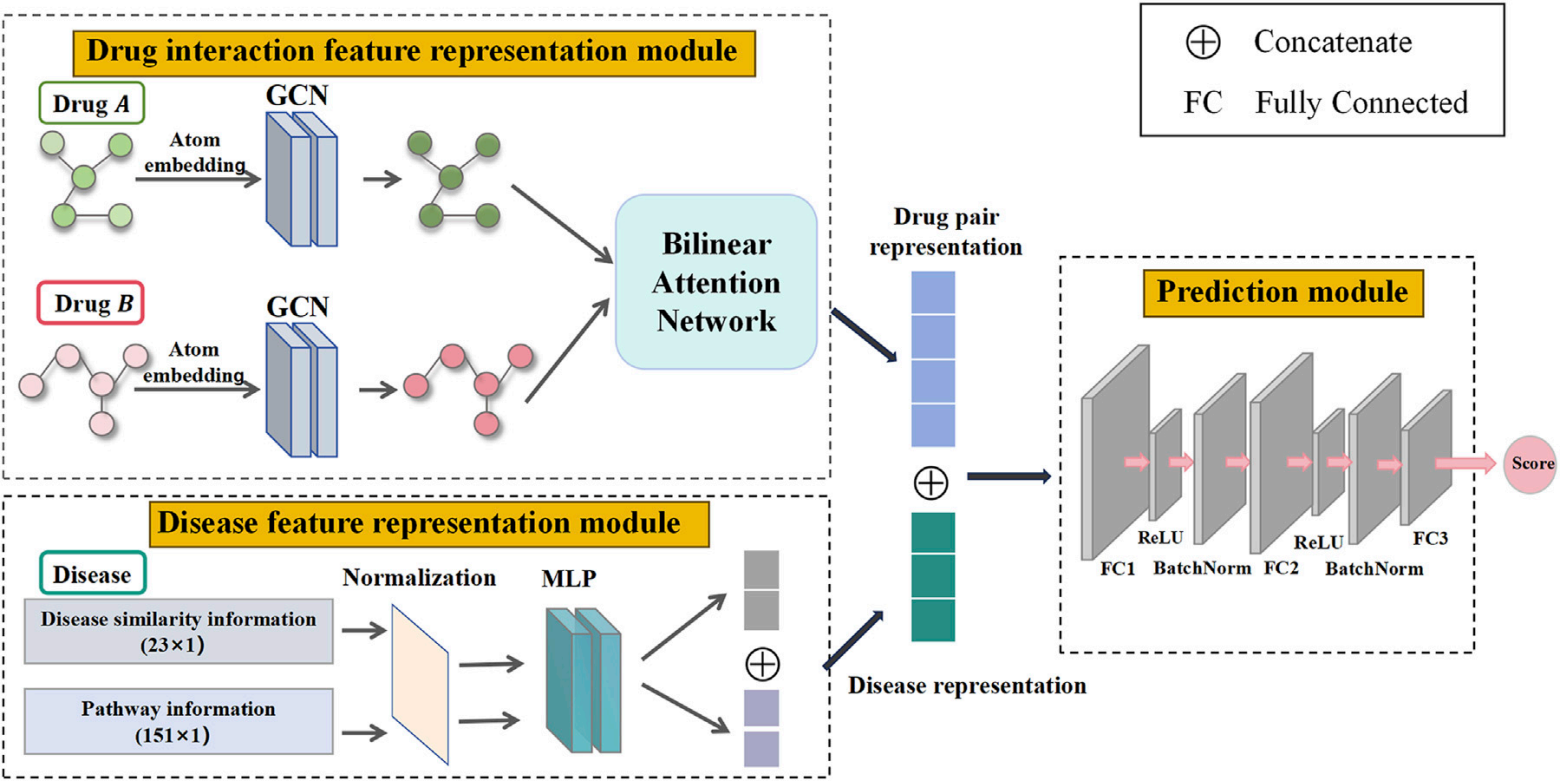
Three modules in TransferBAN-Syn. Three key modules in TransferBAN-Syn are the drug interaction feature representation module, the disease feature representation module, and the prediction module. The drug interaction feature representation module uses GCN to extract atomic-level features from drug molecular graphs and employs a bilinear attention network to capture interactions between drugs, thereby forming a characteristic representation of the drug combination. The disease feature representation module integrates disease pathway and disease similarity information to form a disease feature representation. The prediction module integrates the drug interaction feature representation and disease feature representation to predict the potential of drug combinations in synergistically treating specific diseases.

The TransferBAN-Syn model consists of three key modules as shown in Figure 2: a drug interaction feature representation module, a disease feature representation module, and a prediction module. Specifically, TransferBAN-Syn is initially trained on the data from 21 parasitic diseases to predict their synergistic drug combinations, serving as a source model with its parameters preserved. Then, the target model is developed by retaining the parameters in the drug and disease feature representation modules from the pre-trained source model, and fine-tuning the prediction module to identify potential drug combinations for echinococcosis.

In particular, the drug interaction feature representation module uses Graph Convolutional Networks (GCN) to extract atomic-level features from individual drug molecules. It then employs a bilinear attention network to combine these single drug features, capturing the interactions among drugs and forming a representation of drug combination features. The disease feature representation module combines pathway information and disease similarity for parasitic diseases, encoding these features using a Multilayer Perceptron (MLP) to obtain the disease representation. Finally, the drug combination features and disease features are merged and propagated through a fully connected layer to predict drug synergy combinations. This module serves as the upper part of the TransferBAN-Syn model, predicting potential drug combinations. The specifics of the TransferBAN-Syn model will be detailed below.

#### 2.2.2 Drug interaction feature representation module

For precise and comprehensive representation of information among drug combinations, TransferBAN-Syn employs molecular graphs to depict each drug within a combination. It utilizes GCN to extract atomic-level features and a bilinear attention network to combine single-drug features, capturing interactions between drugs of the drug combination.

This study uses the open-source cheminformatics software RDKit to convert SMILES to molecular graph G , where nodes in the molecular graph represent atoms and edges represent chemical bonds between atoms. Employing DGL-LifeSci python packages (Li et al., 2021), the TransferBAN-Syn model extracts chemical properties of drugs and initializes information for atomic nodes in drug molecular graph G , where each atomic node is represented by a 74-dimensional integer vector detailing eight categories of information: type of atom, degree of atom, count of implicit hydrogens, formal charge, number of free radical electrons, hybridization of atom, count of hydrogens, and the aromatic status of the atom. $\Psi_{\text{d}}$ is utilized to represent the maximum number of atomic nodes in drug molecular graph. If the number of nodes in a drug molecular graph fall below $\Psi_{\text{d}}$ , the matrix is zero-padded to facilitate computations with virtual nodes. Consequently, the node feature matrix of each drug molecular graph is represented as $H_{\mathrm{init}}\;\in R^{\Psi_{d}\times 74}$ . Moreover, a simple linear transformation is used to define $H^{\left(0\right)}=W_{0}{H_{\mathrm{init}}}^{T}$ , resulting in a real-valued dense matrix $H^{\left(0\right)}\in R^{\Psi_{d}\times C}$ as the input feature, where C is the feature representation dimension of the obtained molecule.

Following the construction of molecular graph $H_{A}^{\left(0\right)}$ for drug A , the potential low-dimensional vector representation of the drug molecular graph is learned using GCN. In GCN, drug representation learning involves message passing between each node and its neighbors. This process uses a message-passing neural network model to learn node representations and their relationships within the molecular graph. Finally, pooling operations create a comprehensive representation of the drug molecule.

TransferBAN-Syn employs a L -layer GCN-block to effectively learn the feature representation of drug compounds, with the feature representation of drug A after l+1 layers shown as Equation 1:

$$H_{A}^{\left(l+1\right)}=\sigma \left({\tilde{D}}^{-\frac{1}{2}}\tilde{E}{\tilde{D}}^{-\frac{1}{2}}H_{A}^{\left(l\right)}W^{\left(l\right)}\right),$$ (1)

where $\tilde{E}=E+I$ , and E represents the adjacency matrix of the molecular graph of drug A , I is the identity matrix, and $\tilde{D}$ represents the degree matrix of adjacency matrix A , $H_{A}^{l}\in R^{\Psi_{d}\times C}$ is the feature representation of drug A at the $l^{th}$ layer, $H_{A}^{\left(l\right)}$ represents the learnable parameters at the $l^{th}$ layer, and σ denotes the activation function. Following the $L^{th}$ layer, the feature representation $H_{A}=H_{A}^{\left(L\right)}$ of drug A is acquired.

**FIGURE 3:**
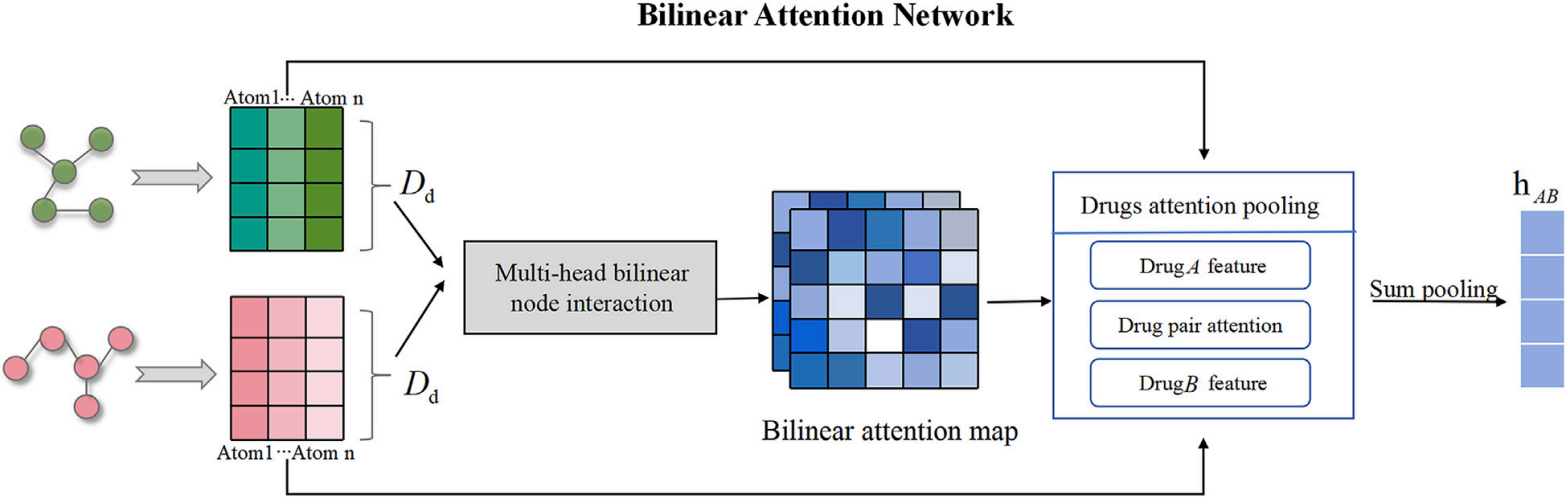
Drug molecular bilinear attention network for extracting drug combination features. The bilinear attention network consists of a bilinear attention step and a bilinear pooling step to generate a joint representation.

TransferBAN-Syn utilizes Bilinear Attention Networks (BAN) to capture the feature representations of pairwise local interactions between drug pairs (as shown in Figure 3). Simultaneously achieving exceptional performance, the time complexity in low-rank bilinear pooling is optimized through matrix chain multiplication and leveraging the attributes of low-rank factorization. Therefore, BAN can capture complex interactions between drug molecules and extract their comprehensive features, which is crucial for understanding and predicting the synergistic effects of drug combinations.

In this research, we apply the BAN module to capture pairwise local interactions between drug combination. The BAN module primarily comprises two components: the construction of the drug bilinear interaction map and the bilinear pooling layer of the drug interaction map, with the former aimed at capturing pairwise attention weights and the latter at extracting the holistic feature representation of the drug combination. If the feature representations $H_{A}$ , $\;H_{B}\in R^{\Psi_{d}\times C}$ of drugs A and B obtained through GCN are to be integrated into an overall feature representation $H_{AB}$ , the first step is to construct the bilinear interaction map $I_{AB}\in R^{C\times C}$ of drugs A and B :

$$I_{AB}=\left[\left(1\cdot q^{T}\right)⊙\sigma \left(H_{A}^{T}\cdot U\right)\right]\cdot \sigma \left(V^{T}\cdot H_{B}\right),$$ (2)

where $U\in R^{\Psi_{d}\times C}$ and $V\in R^{\Psi_{d}\times C}$ are the learnable weight matrices for the features of drugs A and B , respectively, $q\in R^{C}$ is a learnable weight vector, $1\in R^{C}$ is a fixed all-ones vector, and ⊙ denotes the Hadamard product. The $I_{AB}$ obtained from Equation 2 represents the interaction strength between the substructural pairs of drugs A and B .

After obtaining the drug bilinear interaction map $I_{AB}$ , the joint feature representation $H_{AB}^{\prime }$ between drugs is obtained by introducing a bilinear pooling layer. In particular, the $k^{th}$ element of $H_{AB}^{\prime }$ , denoted as $H_{AB\left(k\right)}^{\prime }$ , is computed shown as Equation 3:

$$H_{AB\left(k\right)}^{\prime }=\sigma {\left(H_{A}^{T}\cdot U\right)}_{\left(k\right)}^{T}\cdot I_{AB}\cdot \sigma {\left(H_{B}^{T}\cdot V\right)}_{\left(k\right)},$$ (3)

where the subscript $\left(k\right)$ denotes the $k^{th}$ column of the matrix, for instance, $U_{\left(k\right)}$ represents the $k^{th}$ column vector of the weight matrix U . It is important to note that the bilinear pooling layer does not introduce any new learnable parameters. The weight matrices U and V are shared with the parameters of the preceding drug interaction graph, which serves to decrease the quantity of parameters and alleviate overfitting. Furthermore, sum pooling is applied to the joint representation vector to acquire a compact representation of drug interaction features, denoted as $H_{AB}$ shown as Equation 4:

$$H_{AB}=SumPool\left(H_{AB}^{\prime },s\right),$$ (4)

where the function $SumPool\left(•\right)$ represents a one-dimensional, non-overlapping sum pooling operation, where denotes the stride, reducing the dimensionality of ${H^{\prime }}_{AB}\in R^{C}$ to $H_{AB}\in R^{\frac{C}{s}}$ .

Additionally, by computing multiple drug bilinear interaction map, we can extend the single pairwise interactions into a multi-head format. The final joint representation vector is the sum of all heads. Since the weight matrices U and V are shared, each additional head only introduces a new weight vector q , making this approach highly efficient.

#### 2.2.3 Disease feature representation module

In this study, disease feature representation is achieved by integrating disease pathway and inter-disease similarity information, aiming to improve the accuracy of drug combination predictions for parasitic diseases. The progression of parasitic diseases is often influenced by multiple biological pathways. This pathway information not only reveals the underlying mechanisms of the disease but also facilitates research implementation and result reproducibility due to its relative ease of access and low-dimensionality. Additionally, considering the similarity between diseases helps identify biological markers and pathways shared by different diseases, thereby enhancing the model’s generalization ability. Thus, selecting parasitic disease pathway information and disease similarity as disease features holds significant theoretical and practical value in enhancing the predictive performance and robustness of drug combinations against echinococcosis.

For this study, pathway information related to parasitic diseases and their similarity scores are collected from the Malacards human disease database (http://www.malacards.org/) (Rappaport et al., 2013). For the construction of the disease pathway feature matrix ${Dis}_{\mathrm{pathway}}$ , we aggregate the associated pathway information for each chosen parasitic disease and integrated this information through a union operation, obtaining a consolidated pathway feature set comprising 151 features. The pathway feature matrix ${Dis}_{\mathrm{pathway}}$ thus formed is a binary matrix, with rows denoting various parasitic diseases and columns indicating specific pathways. In the matrix, if an element ${Dis}_{\mathrm{pathway}}\left(i,j\right)$ is 1, it indicates that the $i^{th}$ parasitic disease is associated with the $j^{th}$ pathway; if 0, the two are unrelated. Furthermore, using the disease similarity scores provided by Malacards, we construct a similarity feature matrix for each disease relative to other diseases, denoted as ${Dis}_{\mathrm{sim}}$ . Then, Z -score normalization is applied to those features for standardizing the range of values.

For in-depth extraction of disease features, TransferBAN-Syn utilizes a Multi-Layer Perceptron (MLP) model comprising two hidden layers, with neurons in these layers transforming and extracting features via nonlinear activation functions. The final feature embedding representation $H_{z}$ for disease z is achieved by extracting pathway and similarity features using MLP and then concatenating them shown as Equation 5

$$H_{z}=MLP\left(Dis_{\mathrm{pathway}}\right)\|MLP\left(Dis_{\mathrm{sim}}\right),$$ (5)

where ‖ denotes the feature concatenation operation.

#### 2.2.4 Prediction module

The prediction module concatenates drug interaction feature and disease feature to ascertain the potential of a drug combination to synergistically treat a specific disease.

The interaction features $H_{AB}$ of drugs A and B are initially concatenated with the feature $H_{z}$ of disease z . Then, a MLP is employed to predict the probability $P_{\left(A,B,z\right)}$ of the combination of drugs A and B in treating disease z , shown as Equation 6:

$$P_{\left(A,B,z\right)}=softmax\left(MLP\left(H_{AB}\|H_{z}\right)\right),$$ (6)

where $softmax\left(•\right)$ refers to the softmax function. If $P_{\left(A,B,z\right)}$ is closer to 1, then it indicates a higher probability of the drug combination A and B in treating disease z .

Finally, all learnable parameters are jointly optimized via backpropagation. To boost the model’s generalization capability, a minimized cross-entropy loss function with L2 regularization is employed shown as Equation 7:

$$L=-\underset{i}{\sum }\left(y_{i}\log\left(p_{i}\right)+\left(1-y_{i}\right)\log\left(1-p_{i}\right)\right)+\frac{\lambda }{2}\|\Theta {\|}_{2}^{2},$$ (7)

where Θ represents the collection of all learnable weight matrices and bias vectors, and λ is the hyperparameter for L2 regularization to control model complexity and prevent overfitting. $y_{i}$ is the true label for the $i^{th}$ pair of drug targets (takes the value of 0 or 1), and $p_{i}$ is the probability output by the model.

## 3 Results

### 3.1 Experimental parameter settings

The TransferBAN-Syn algorithm is implemented in a Python 3.8 and PyTorch 1.7.1 environment. In the algorithm configuration, the batch size is set to 64. The maximum number of atoms in drug molecules is set to 150. The embedding dimension C is set to 384, and the stride s for bilinear pooling is set to 3.

The architecture of TransferBAN-Syn is essentially determined by a set of hyperparameters, including the GCN layers, learning rate, activation function, the hierarchical structure of training rounds, and so on. Considering the computational cost of exhaustively enumerating hyperparameters, we adopted a grid search strategy to adjust these parameters. Details of the parameter adjustments are provided in Supplementary Table S2. The selection of hyperparameters is refined through five-fold cross-validation on a benchmark dataset. The experimental results, detailed in the supplementary materials, demonstrate that the optimal configuration for the GCN involves three layers with dimensions [128, 256, 128], which effectively extract drug features as shown in Supplementary Figure S1. In the MLP used for disease feature extraction, the best performance is achieved with two hidden layers sized [128, 256]. The multi-head bilinear attention mechanism performs optimally with two heads as shown in Supplementary Figure S2. Additionally, the fully connected prediction layer includes 512 hidden units. The ReLU activation function is selected to enhance model performance, and the learning rate for the optimizer is set at 5e-5 as shown in Supplementary Figure S3. Subsequent experiments are conducted using these optimized model parameters.

### 3.2 Baseline methods

To evaluate the predictive performance of the TransferBAN-Syn model, we compared it with five state-of-the-art predicting drug synergy combinations, including classic machine learning and deep learning-based methods such as TreeCombo (Janizek et al., 2018), DeepSynergy (Preuer et al., 2018), TranSynergy (Liu and Xie, 2021), GAECDS (Li et al., 2023), and Attensyn (Wang et al., 2023).

• TreeCombo: This is an XGBoost-based algorithm designed to predict the synergy of drug combinations using drug properties and gene expression levels. TreeCombo can effectively uncover complex nonlinear relationships between drug features and synergistic effects.

• DeepSynergy: This is a deep learning model designed to predict drug combination synergy. DeepSynergy processes concatenated input vectors representing two drugs and one cell line through multiple hidden layers to produce a synergy score. It employs data normalization techniques and hyperparameter tuning to optimize predictive performance. The model’s architecture includes conic layers and dropout regularization to improve generalization and accuracy.

• TranSynergy: This is a deep learning model that predicts drug combination synergy by integrating gene dependency, gene-gene interaction, and drug-target interaction profiles. The model uses a transformer component with self-attention mechanisms to encode gene-gene interactions. Drug and cell line features are represented by a combination of gene expression and dependency data, which are processed through neural networks to predict synergy scores.

• GAECDS: It is an algorithm that predicts drug synergy using a combination of a graph autoencoder and CNN. The GAE module encodes drug features and synergistic relations into latent vectors, which are then reconstructed to predict new synergistic relations. These encoded features are combined with cell line data and processed by the CNN module to predict the synergy scores of drug combinations.

• Attensyn: It is an attention-based deep graph neural network designed to predict the synergy of anti-cancer drug combinations. The algorithm converts drug SMILES strings into molecular graphs, and utilizes a graph-based drug-embedding module with GCN and LSTM layers to extract multi-resolution features. An attention-based pooling module learns interactive information between drug pairs and identifies important chemical substructures, which are then fed into a fully connected neural network for synergy prediction.

### 3.3 Performance evaluation

This study assesses the performance of our TransferBAN-Syn model by comparing it with the aforementioned five state-of-the-art drug synergy combination prediction methods. To fairly compare the algorithms, we apply the same transfer learning strategy to all five algorithms and ours. Specifically, the drug combination prediction tasks for 21 other parasitic diseases are selected as the source tasks, with the drug combination prediction task for echinococcosis serving as the target task. The predictive capacities of the models are assessed using five iterations of five-fold cross-validation, wherein the training samples are randomly divided into five roughly equal subsets, with one subset reserved as the test set for each iteration and the remainder serving as the training set. During our experiments, we encountered an imbalance between the positive and negative samples in the target domain, where the ratio of positive to negative samples was approximately 1:2. This imbalance posed a challenge to the modelâ€™s predictive accuracy and generalization ability. To address this issue, we implemented an under-sampling technique in the target domain. Specifically, for each iteration, we randomly selected a subset of negative samples that matched the number of positive samples, effectively balancing the dataset. The average predictive accuracy from the five iterations of five-fold cross-validation serves as the ultimate metric for performance evaluation. The performance evaluation metrics include Area Under the Curve (AUC), the Area Under the Precision-Recall curve (AUPR), Recall (Rec), Precision (Prec), F1 score (F1), and accuracy (ACC) to comprehensively reflect the model’s performance in various aspects. The experimental results are shown in Table 2.

**TABLE 2:** Results (Mean ± STD) of TransferBAN-Syn and other five state-of-the-art drug synergy combination prediction methods in terms of six classification metrics.

	AUC	AUPR	Rec	Prec	F1	ACC
TreeCombo	0.9287 ± 0.03	0.8960 ± 0.03	0.8333 ± 0.02	0.8630 ± 0.02	0.8694 ± 0.02	0.8778 ± 0.03
Deepsyn	0.9180 ± 0.01	0.9378 ± 0.03	0.9087 ± 0.02	0.8526 ± 0.01	0.8723 ± 0.02	0.8407 ± 0.03
Transyn	0.9340 ± 0.02	0.9472 ± 0.02	0.9105 ± 0.03	0.8763 ± 0.02	0.8794 ± 0.02	0.8772 ± 0.02
GAECDS	0.9196 ± 0.02	0.9429 ± 0.01	**0.9284 ± 0.01**	0.8928 ± 0.03	0.8619 ± 0.02	0.8534 ± 0.02
Attensyn	0.9425 ± 0.01	0.9537 ± 0.01	0.8334 ± 0.01	0.8848 ± 0.02	0.8983 ± 0.02	0.8630 ± 0.01
TransferBAN-Syn	**0.9561 ± 0.01**	**0.9622 ± 0.01**	0.9142 ± 0.01	**0.9115 ± 0.01**	**0.9304 ± 0.01**	**0.9220 ± 0.02**

The results indicate that TransferBAN-Syn performs outstandingly across all metrics, demonstrating excellent stability and robustness under different data splits. Specifically, TransferBAN-Syn has a precision of 0.9115, the highest among all models, indicating the lowest false positive rate in predicting positive samples. Its accuracy is 0.9220, also the highest, showing the best overall classification accuracy across all samples. Although TransferBAN-Syn’s recall is 0.9142, second only to GAECDS’s 0.9284, it still performs excellently, indicating the model’s effectiveness in identifying most positive samples. GAECDS combines graph autoencoder (GAE) and CNN, capturing complex relationships between drugs through the GAE module and performing collaborative score prediction through the CNN module. This combined structure allows for a more comprehensive handling and integration of various features, thereby enhancing the model’s ability to predict positive samples. However, the GAECDS model may overfit to positive samples during training, resulting in an inability to effectively distinguish negative samples, thereby reducing prediction accuracy and the performance of AUC and AUPR values. The results indicate that TransferBAN-Syn’s F1 score is 0.9304, the best among all algorithms, showing that the model excels in balancing precision and recall, making it the model with the best overall performance.

Overall, the TransferBAN-Syn model not only exhibits high prediction accuracy but also demonstrates excellent stability and robustness.

### 3.4 Ablation study

**FIGURE 4:**
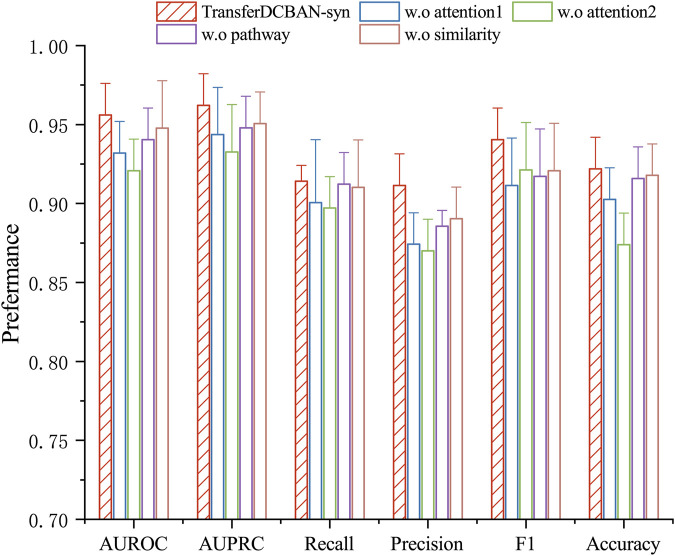
Ablation study results for TransferBAN-Syn. The lines represent the positive error bars of the standard deviation.

To investigate the impact of the transfer learning strategy, bilinear attention module, and disease feature representation on model performance, we conduct a series of ablation experiments testing various variants of the TransferBAN-Syn algorithm. (1) The original TransferBAN-Syn model; (2) A model that removes the bilinear attention module in favor of a self-attention module (w.o attention1); (3) A model that removes the attention module and directly predicts (w.o attention2); (4) A model without disease pathway information (w.o pathway); (5) A model excluding disease similarity information (w.o similarity); and (6) A model that does not utilize transfer learning from other parasitic disease information (w.o Transfer). The experimental results are shown in Figure 4.

The experimental results show that the original TransferBAN-Syn model performs the best. As shown in Figure 4, feature fusion with attention mechanisms, as opposed to direct concatenation of drug features, better captures the higher-order features of drug combinations. Compared to traditional self-attention networks, the bilinear attention module can more effectively capture the interactions between drug combinations, particularly the pairwise interaction information between their substructures, which may be the main reason for the superior performance of the original model. Moreover, the pathway information of diseases plays a significant role in providing accurate disease feature representations.

**FIGURE 5:**
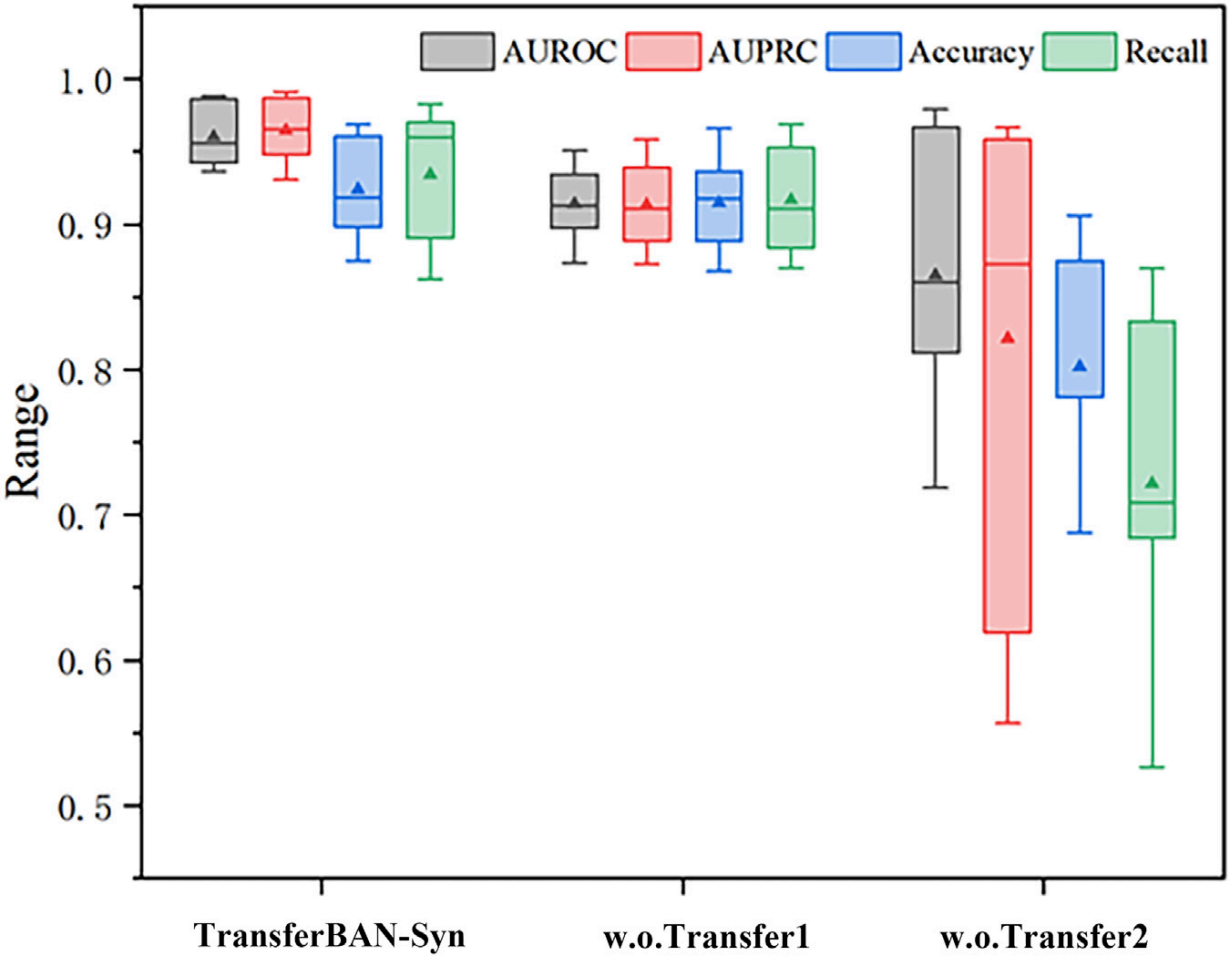
Efficacy analysis of Transfer Learning in TransferBAN-Syn The box plots show the median as the center lines, and the mean as the triangles.

It is noteworthy that without using information from other parasitic diseases for transfer learning, the model struggles to be effectively trained with only echinococcosis data, indicating that leveraging information from other parasitic diseases for auxiliary training is effective and necessary in data-scarce scenarios of predicting drug combinations against echinococcosis. These findings emphasize the importance of each module in the model for enhancing predictive performance and confirm the effectiveness of integrating various sources of information and model structures in predicting drug combinations against echinococcosis. More importantly, through Table 3, we can see that the introduction of the transfer learning strategy in the results has the most significant improvement in experimental outcomes. In the TransferBAN-syn model, the introduction of transfer learning techniques significantly enhances the model’s predictive capability and stability. Specifically, we implemented a variant—referred to as w. o transfer1—that trains the model simultaneously on both the Echinococcosis data and the 21 parasitic diseases data, with the fine-tuning step removed. We also evaluated a model trained solely on Echinococcosis data without employing the transfer learning framework, referred to as w. o transfer2. From the experimental results presented in Figure 5, we observe that our proposed TransferBAN-Syn model outperforms both w. o transfer1 and w. o transfer2 in terms of predictive accuracy and stability. Specifically, while the w. o transfer1 shows some improvement over training solely on Echinococcosis data (w.o transfer2), it does not achieve the same level of performance as our transfer learning model. This indicates that simply combining data from Echinococcosis and other parasitic diseases without proper knowledge transfer is insufficient. This ablation study reveals the key finding that integrating information related to 21 types of parasitic diseases can significantly improve the performance of the echinococcosis prediction model, while the transfer learning strategy effectively utilizes data from 21 parasitic diseases similar to echinococcosis, further enhancing the precision of echinococcosis predictions. Therefore, the application of the transfer learning strategy can not only effectively mine deep information related to echinococcosis but also play an important role in improving the accuracy of drug combination predictions.

**TABLE 3:** Synergistic effects and scores of different drug combinations for different disease types.

Drug A	Drug B	Disease-type	Synergistic effects	Score
Albendazole	Harmine	AE	Y	0.9997
Albendazole	Verapamil	CE	Y	0.9996
Albendazole	Harmine	CE	Y	0.9995
Albendazole	Carvacrol	AE	Y	0.9993
Flubendazole	Nitazoxanide	CE	Y	0.9993
Piperacillin	Albendazole	AE	N	0.0057
Mebendazole	Ticarcillin	CE	N	0.0048
Mebendazole	Carbenicillin	AE	N	0.0035
Mebendazole	Piperacillin	CE	N	0.0027
Mebendazole	Cisplatin	AE	N	0.0024

### 3.5 Case study

Based on the performance evaluation experimental results of this study, the proposed the TransferBAN-syn model demonstrated significant superior performance in predicting echinococcosis drug combinations. To verify the model’s generalization ability, the work employs an independent validation set to assess the model’s predictive accuracy. Specifically, from the collected echinococcosis drug combination dataset, we select five drug combinations known to have synergistic effects and five drug combinations without synergistic effects, and set them as an independent test set to test the model’s discrimination ability. The remaining drug combination data are used to fine-tune the pre-trained general model, we retain the best-performing model parameters and tested them on the independent validation set. The test results, shown in Table 3, reveal that the trained model can not only accurately identify drug combinations with synergistic effects (positive samples) but also effectively distinguish drug combinations without synergistic effects (negative samples). These results strongly suggest that our model possesses high accuracy and discriminative ability in predicting the synergistic effects of drug combinations, providing powerful tools and methodological guidance for future research in this field.

In order to thoroughly investigate the application of the TransferBAN-syn model in predicting the efficacy of new drug combinations, this study devise an independent validation set approach to precisely evaluate the model’s capability to predict unknown drug combinations. The study reveal through external validation the effectiveness of the model in identifying drug combinations that potentially have therapeutic potential for alveolar echinococcosis (AE) and cystic echinococcosis (CE). All potential drug combinations are systematically arranged and screened from the database, excluding those known to have synergistic effects on AE or CE, thereby yielding a set of potential therapeutic combinations. The synergy probabilities of these combinations are calculated and ranked through the model, identifying ten most promising synergistic drug combinations for AE and CE, respectively, with the detailed lists shown in Tables 4, 5. We have conducted a literature-based validation of the predicted drug combinations. For example, the combination of Primaquine and Pyronaridine Tetraphosphate is supported by existing research, which shows that these drugs exhibit synergistic effects in the treatment of malaria. Primaquine generates reactive oxygen species that disrupt the parasite’s mitochondrial function, while Pyronaridine Tetraphosphate inhibits heme detoxification in the parasite (Stone et al., 2022). The complementary mechanisms of these drugs suggest that they could also have potential as a synergistic combination for treating echinococcosis. These findings not only provide important guidance for the design of future drug combination treatment schemes but also open new possibilities for the development and validation of new drugs, as well as offer new research directions and theoretical bases for subsequent biological experimental designs and echinococcosis treatment studies.

**TABLE 4:** Potential synergistic drug combinations for alveolar echinococcosis evaluated by TransferBAN-Syn.

Drug A	Drug B	Disease-type	Score
Oxytetracycline	Lamotrigine	AE	0.9998
Fulvestrant	Bifendate	AE	0.9998
Suramin	Vancomycin	AE	0.9997
Arachidonic Acid	Pyronaridine Tetraphosphate	AE	0.9995
Emodepside	Pentamidine	AE	0.9994
Clotrimazole	Terfenadine	AE	0.9993
Clemastine	Pyronaridine	AE	0.9993
Thymosin	Pyronaridine Tetraphosphate	AE	0.9993
Emodepside	Rimonabant	AE	0.9992
Imidocarb Dipropionate	Rifampin	AE	0.9990

**TABLE 5:** Potential synergistic drug combinations for cystic echinococcosis evaluated by TransferBAN-syn.

Drug A	Drug B	Disease-type	Score
Clotrimazole	Terfenadine	CE	0.9997
Rifapentine	Octreotide	CE	0.9996
Piperaquine	Sophoraisoflavone A	CE	0.9996
Primaquine	Pyronaridine Tetraphosphate	CE	0.9994
Raloxifene	Bifendate	CE	0.9993
Thymosin	Imiquimod	CE	0.9993
Dipyridamole	Colchicine	CE	0.9993
Thymosin	Eprinomectin	CE	0.9992
Halofantrine	Pyronaridine Tetraphosphate	CE	0.9991
Itraconazole	Terfenadine	CE	0.9991

## 4 Discussion and conclusion

Echinococcosis, as a chronic and complex parasitic disease, poses a serious threat to human health. Pharmacotherapy is indispensable in the treatment of echinococcosis, with combination drug therapy demonstrating higher treatment efficacy and lower risk of drug resistance. However, given the unique complexity of echinococcosis and the scarcity of treatment drug combination data, discovering effective drug combinations becomes particularly challenging. In response to this challenge, the TransferBAN-Syn model propose in this paper adopts a transfer learning strategy, supplementing echinococcosis data with drug combination data from other parasitic diseases. This strategy not only enhances the model’s accuracy in predicting the synergistic effects of echinococcosis drug combinations but also improves the model’s generalizability. Furthermore, by constructing a drug combination dataset for 21 parasitic diseases, this paper further enriches the research foundation, providing valuable resources for subsequent drug discovery and evaluation. Additionally, the TransferBAN-Syn model effectively captures the complex interactions between drug molecules through deep graph neural networks and attention-based aggregation modules, thereby achieving accurate prediction of drug combination synergistic effects. Compared to five state-of-the-art traditional machine learning methods and deep learning models, the TransferBAN-Syn model has shown significant performance advantages, proving its potential application in the study of drug combinations against echinococcosis.

Future studies will aim to further refine the algorithm’s framework, reduce computational complexity, and explore more effective transfer learning strategies to better address differences among various parasitic diseases. Moreover, expanding the drug combination dataset to include a broader range of parasitic diseases and drug combinations will enhance the model’s generalizability and applicability. With these improvements, we hope to more accurately and efficiently predict and evaluate the synergistic effects of drugs against echinococcosis in the future, providing a more reliable scientific basis for the treatment of echinococcosis.

## Data Availability

The original contributions presented in the study are publicly available. This data can be found here: https://github.com/ahu-bioinf-lab/TransferBAN-Syn.
