# Supplementary material for: TransferBAN-Syn: a transfer learning-based algorithm for predicting synergistic drug combinations against echinococcosis
Source: Front Genet. 2025 Jan 6;15:1465368. doi: 10.3389/fgene.2024.1465368 (PMC11743481; doi:10.3389/fgene.2024.1465368)
Supplement: Supplementary file 1 [file Presentation1.pdf]

SUPPLEMENTARY MATERIALS

0.1 21 Parasitic Diseases

Table S1. List of 21 Parasitic Diseases Used in TransferBAN-Syn

Number	Parasitic disease
1	Malaria
2	Cysticercosis
3	Ancylostomiasis
4	Filariasis
5	Trichuriasis
6	Schistosomiasis
7	Myiasis
8	Strongyloidiasis
9	Giardiasis
10	Amebiasis
11	Fascioliasis
12	Ascaridiasis
13	Toxocariasis
14	Trichinosis
15	Chagas Disease
16	Trypanosomiasis
17	Mucocutaneous Leishmaniasis
18	Cutaneous Leishmaniasis
19	Visceral leishmaniasis
20	Onchocerciasis
21	Elephantiasis

0.2 Hyperparameter Settings

In the hyperparameter sensitivity analysis, the actual architecture of Transfer-BAN is determined by hyperparameter settings, including learning rate, activation function, number of training epochs, and the number of layers and units in GCN and MLP. Since it is computationally infeasible to exhaustively search all hyperparameter combinations, we use a grid search method to adjust hyperparameters. As shown in Table S1, we tested different structural forms and values of these hyperparameters. We tuned the hyperparameters through five-fold cross-validation on the benchmark dataset, with the final selected values highlighted in bold.

The optimal number of GCN layers L is determined to be 3 through parameter selection experiments, as shown in Figure S1. We believe that when the number of layers is less than 3, the features are not sufficiently aggregated; and when the number of layers exceeds 3, over-smoothing occurs, leading to a decline in classification performance.

The GCN structure, consisting of three hidden layers with dimensions [128,256,128], yields the best performance in drug feature extraction. We also considered different numbers of hidden layers. For the MLP model extracting disease similarity features, hidden layers [64,128] performed best, while for the MLP extracting disease pathway features, hidden layers [128,128] yielded the best performance.

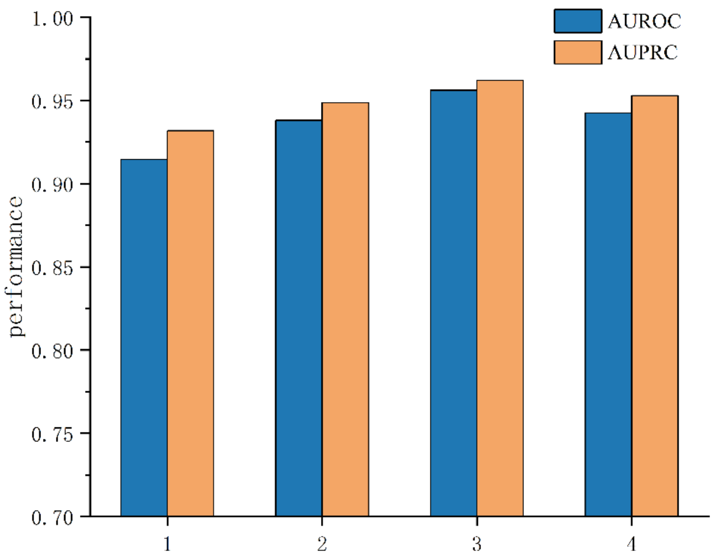

Figure S1: **The Impact of Different Numbers of GCN Layers on Model Prediction Performance.** TransferBAN-Syn consists of source domain and target domain models. The source domain model is pre-trained with data-rich parasitic diseases to comprehend the underlying mechanisms between drug combinations and diseases. The target domain model for echinococcosis shares parameters with the source domain model and fine-tunes the prediction module parameters to achieve optimal predictive performance.

For the multi-head bilinear attention mechanism, we evaluated multiple distinct values and found that with 2 attention heads, a latent embedding size k of 256 for drug pairs, and a sum pooling window size s of 3, the performance was optimal. After multiple evaluations, we selected [256,128] as the number of hidden neurons in the fully connected predictor. In practice, the learning rate and the number of attention heads in the source and target tasks significantly impact model performance, while the dropout rate and other hyperparameter values have relatively minor effects. For the activation function, we chose to use ReLU.

Table S2. Hyperparameter Settings for the Transfer-BAN Model

**Hyperaramater**	**Values**
GCN hidden uints	[128,128];[128,256];[256,256];[128,128,128];**[128,256,128]**
MLP hidden uints(Disease similarity)	[64,64];**[64,128]**;[128,128];[128,256]
MLP hidden uints(Disease pathway)	**[128,128]**;[128,256];[256,128];[256,256]
MLP hidden uints(Predictor)	[256,256];**[256,128]**;[256,512];[256,128,128];[256,512,128]
BAN Head	**2**,4,6,8
k	64;128;**256**;512
s	2,**3**,4,5
L	1,2,**3**,4
Learning rate(Source Data)	5e-3; 5e-4; **5e-5**;5e-6
Learning rate(Target Data)	5e-5; 5e-6; **5e-7**
Dropout	No dropout;0.1;0.2;**0.3**;0.4;0.5

Additionally, we experimented with different numbers of attention heads in the model and found that having two heads yielded the best overall performance, as shown in Figure S2. Furthermore, we experimented with different learning rates for different source domains, finding that a learning rate of 10e-5 yielded the best overall performance, as shown in Figure S3.

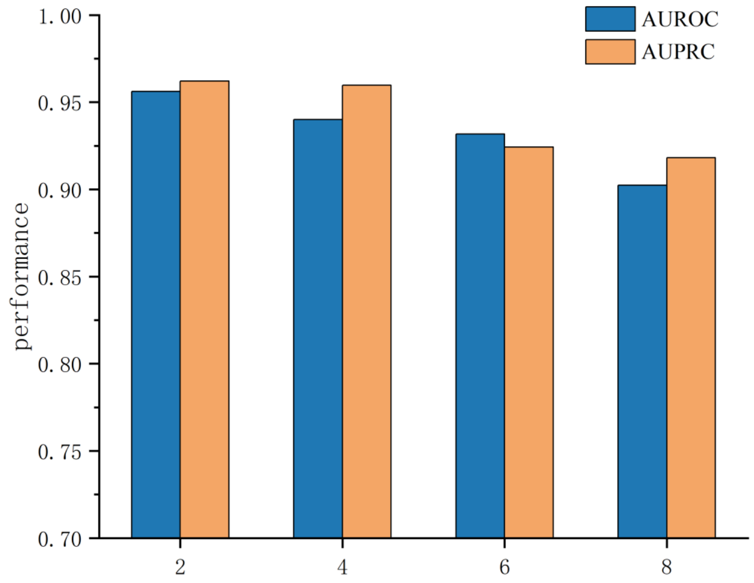

Figure S2: The Impact of Different Numbers of Attention Heads on Model Prediction Performance.

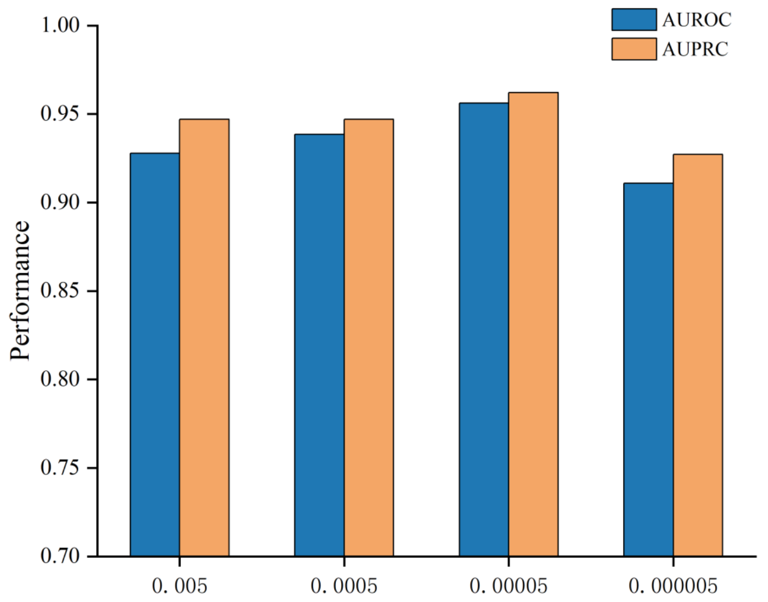

Figure S3: The Impact of Different Learning Rates on Model Prediction Performance.
